# Targeting Myeloid-Derived Suppressor Cells Is a Novel Strategy for Anti-Psoriasis Therapy

**DOI:** 10.1155/2020/8567320

**Published:** 2020-06-28

**Authors:** Chao Chen, Lirong Tan, Wu Zhu, Li Lei, Yehong Kuang, Panpan Liu, Jie Li, Xiang Chen, Cong Peng

**Affiliations:** ^1^Department of Dermatology, Xiangya Hospital, Central South University, Changsha, Hunan, China; ^2^Hunan Key Laboratory of Skin Cancer and Psoriasis, Xiangya Hospital, Central South University, Changsha, Hunan, China; ^3^Hunan Engineering Research Centre of Skin Health and Disease, Xiangya Hospital, Central South University, Changsha, Hunan, China; ^4^National Clinical Research Center for Geriatric Disorders, Xiangya Hospital, Central South University, Changsha, Hunan 410008, China

## Abstract

Psoriasis is a common immune-mediated, chronic inflammatory genetic-related disease that affects patients' quality of life. Myeloid-derived suppressor cells (MDSCs) are a heterogeneous population of progenitor and immature myeloid cells which are expanded in psoriatic skin lesions and peripheral blood. However, the role of MDSCs in the pathogenesis of psoriasis remains unclear. Here, we confirmed that the accumulation of human MDSCs is remarkably increased in skin lesions of psoriasis patients by flow cytometry. Depleting MDSCs by Gemcitabine significantly suppresses IMQ-induced psoriatic inflammation and epidermal thickening as well as Th17 and Treg cell accumulation. Moreover, through the RNA-Seq technique, we validated some differentially expressed genes on CD4^+^ T-cells of IMQ-induced-MDSC-depleted mice such as IL-21 and Timd2, which are involved in Th17-cell differentiation or T-cell activation. Interestingly, neutralizing IL-21R by antibody reduces IMQ-induced epidermal thickening through downregulating the infiltration of MDSCs and Th17 cells. Our data suggest that targeting myeloid-derived suppressor cells is a novel strategy for antipsoriasis therapy. IL-21 may be a potential therapeutic target in psoriasis.

## 1. Introduction

Psoriasis is a common immune-mediated, chronic inflammatory skin disease, which has been characterized by epidermal acanthosis, hyperkeratosis, parakeratosis, and extensive inflammatory cell infiltration including T-lymphocytes, macrophages, mast cells, and neutrophils [1]. Accumulating evidence showed that the psoriatic keratinocytes (KCs) not only have been shown uncontrolled proliferation but also respond to cytokines such as IL-22 or IL-17A/IL-17F released from Th17 or Th22 cells, which facilitate the secretion of proinflammatory factors such as AMP activating dendritic cells to initiate specific T-cell-related immune responses [1, 2]. More importantly, psoriatic KCs recruit immune cells into psoriatic skin lesions through the production of chemokines or cytokines including myeloid-derived suppressor cells (MDSCs) [3–6].

MDSCs (myeloid-derived suppressor cells) are a heterogeneous population of progenitor and immature myeloid cells, which have been generated during a variety of pathologic conditions such as cancer, infectious diseases, and autoimmune disorders [7–9]. Murine MDSCs are characterized by coexpression of CD11b and Gr-1, whereas human MDSCs are most commonly identified by CD11b^+^ and CD33^+^ with low levels of HLA-DR, the major histocompatibility complex (MHC) class II molecule [7, 10]. MDSCs consist of two large groups of cells: granulocytic or polymorphonuclear MDSCs (PMN-MDSCs, CD11b^+^CD14^−^CD15^+^CD33^+^HLA-DR^−/lo^) and monocytic MDSCs (M-MDSCs, CD11b^+^CD14^+^CD15^−^CD33^+^HLA-DR^−/lo^) [9]. Moreover, it was reported that CD14^+^HLA-DR^−/lo^ monocytic MDSCs are more suppressive than PMN-MDSCs and have emerged as important mediators of tumor-induced immunosuppression [9, 11].

In normal conditions, MDSCs have differentiated into mature granulocytes, macrophages, or dendritic cells (DCs) in bone marrow [9]. However, under pathological conditions such as cancer, chronic inflammatory diseases, and immune diseases, those undifferentiated immature myeloid cells have been recruited and infiltrated into the specific organ from bone marrow [7]. Although MDSCs have been shown a remarkable ability to suppress T-cell responses in cancer, it becomes more heterogeneous and complicated in autoimmune diseases. Recent studies revealed that expanded MDSCs induce immune responses in systemic lupus erythematosus (SLE), autoimmune arthritis (RA), and autoimmune encephalomyelitis [12–15]. Interestingly, studies showed that the population of MDSCs has been expanded in psoriasis patients, which produce cytokines including IL-23, IL-1*β*, and CCL4 [16–18]. Moreover, MDSCs isolated from psoriasis patients fail to suppress T-cell activation and express reduced programmed cell death protein-1 (PD-1), as a consequence of losing the ability to induce regulatory T-cell conversion compared with those cells from healthy controls or melanoma patients [16, 19], indicating MDSCs showed great heterogeneity under different pathogenesis.

In this study, we aim to investigate the proinflammatory roles of MDSCs in the pathogenesis of psoriasis. We found it is a novel strategy to target myeloid-derived suppressor cells for antipsoriasis therapy.

## 2. Materials and Methods

### 2.1. Human Skin Samples

This study was reviewed and approved by the local ethics Institutional Review Board (IRB) (Xiangya Hospital, Central South University, IRB-201512526). All experiments were conducted in accordance with the Declaration of Helsinki Principles. We performed a cross-sectional study of 27 patients with psoriasis and 17 healthy control subjects without inflammatory skin disease. Inclusion criteria included psoriasis patients or healthy control subjects older than 18 years of age, able to give written informed consent, and able to give skin samples. Exclusion criteria included patients on subcutaneous and intravenous systemic immunosuppressant medications. Patients were clinically evaluated for psoriasis subtype and PASI score.

### 2.2. IMQ-Induced Psoriasis-Like Skin Inflammation

Six- to eight-week-old mice were treated with daily topical doses of 62.5 mg of IMQ cream (5%, 3.125 mg of the active compound; Aldara, 3M Pharmaceuticals), which was applied to their shaved backs for 6 consecutive days. A scoring system based on the clinical Psoriasis Area and Severity Index (PASI) was used to evaluate the skin inflammation on the skin lesions of mice. Briefly, erythema, scale, and infiltration were graded on a scale from 0 to 4 as follows: 0, none; 1, slight; 2, moderate; 3, marked; and 4, very marked. The level of erythema was scored using a table with red taints. The cumulative score served as a measure of inflammation severity (scale: 0–12) [20]. The animal study protocol was approved by the Ethics Committee of Xiangya Hospital (Central South University, China, #2015110134).

### 2.3. In Vivo Treatments

Gemcitabine treatment: BALB/c mice were injected intraperitoneally with Gemcitabine (Selleckchem, Houston, TX, USA) on days -1, 1, and 3 at the dose of 40 mg/kg; IMQ was applied from day 1 to their shaved backs for 5 consecutive days topically. The mice were photographed and sacrificed for skin lesion analysis on day 8 (mice divided into 3 groups: vehicle (IMQ+vehicle), Gemcitabine (IMQ+GEM), and untreated (normal)). Anti-IL-21R antibody treatment: BALB/c mice were injected intraperitoneally with anti-mouse IL-21R antibody (4A9) (BioXCell, West Lebanon, NH, USA) on days -2, 0, 1, 3, and 5 by i.p. injection of 140 *μ*g anti-IL-21R antibody; IMQ was applied from day 1 to their shaved backs for 6 consecutive days topically. The mice were photographed and sacrificed for skin lesion analysis on day 8 (mice divided into 3 groups: vehicle (IMQ+vehicle), anti-IL-21R antibody (IMQ+Anti-IL-21R), and untreated (normal)).

### 2.4. Tissue Processing

Skin lesions of psoriasis patient or mice were cut into small pieces and digested in 5 ml PBS containing 2 mg/ml collagenase type IV and 1 mg/ml dispase II (both Sigma-Aldrich, USA) while shaking at 37°C for 150 minutes. Enzyme activity was stopped using 10% FBS medium. The tissue was further homogenized with a syringe and filtered through a 40 *μ*m cell strainer. The cell strainer was washed with 20 ml PBS followed by centrifugation (500 x g at 4°C for 10 min). Single-cell suspensions from the spleens were obtained by mashing the spleens through 40 *μ*m cell strainers. The cell strainer was washed with 20 ml PBS followed by centrifugation (500 x g at 4°C for 5 min) and then split red blood cells by means of lysing solution (BD Pharm Lyse™, USA). Single cells were then stained with fluorescence antibodies for flow cytometry.

### 2.5. Flow Cytometry

All utilized antibodies are summarized in Supplementary Table S2. Firstly, Zombie Aqua™ Fixable Viability Dye was used for selecting living cells. Then, TruStain fcX anti-mouse CD16/32 was used to block Fc receptor on the immune cells of mice. For surface staining, single cells isolated from the skin or the spleens were incubated with antibodies at 4°C for 30 min, followed by washing and centrifugation (500 x g at 4°C for 5 min). For intracellular cytokine staining (Th17), cells were restimulated in 100 *μ*l RPMI supplemented with GolgiPlug (1 : 1000, BD), PMA (50 ng/ml, AppliChem), and ionomycin (750 ng/ml, Invitrogen) for 4 to 6 hours at 37°C. After surface staining, cells were permeabilized and fixed in 250 *μ*l BD Cytofix/Cytoperm™ according to the manufacturer's instructions. Then, the cells were washed with permeabilization buffer and stained intracellularly at 4°C for 30 min in the permeabilization buffer. For intranuclear staining (Tregs), after surface staining, cells were fixed and permeabilized using the eBioscience Foxp3/transcription factor fixation/permeabilization concentrate and diluent from ThermoFisher followed by incubation with anti-mouse/rat Foxp3 antibodies at room temperature for 40 min according to the manufacturer's instructions. To better distinguish the border between positive and negative subsets, we set FMO-controls for markers including IL-17A, IFN-*γ*, CD25, and Foxp3. The acquisition was performed with FACS Canto II (BD Biosciences). Flow cytometric analysis on live, single cells was performed using FlowJo (Tree Star) software.

### 2.6. Quantitative RT-PCR (qRT-PCR)

Total RNA was extracted with Trizol (Invitrogen), and cDNA was synthesized via reverse transcription using a HiScript Q RT Kit (Vazyme) (R123-01). qRT-PCR was performed using an UltraSYBR Mixture with ROX (CWBio, Beijing, China) according to the manufacturer's instructions on a QuantStudio 3 RT-PCR instrument (ThermoFisher, USA). The reaction mixture contained 0.5 ml of forward and reverse mouse primers, as described in Supplementary Table S1. Values were normalized to Gapdh. All reactions were conducted in triplicate across. Relative quantification was performed using the *ΔΔ*CT method, and the results were expressed in a linear form using the formula 2^−*ΔΔ*CT^.

### 2.7. Cell Sorting for RNA Sequencing

Splenic cells were isolated from the freshly obtained spleen of mice. CD4^+^ T-cells were positively selected from splenic cells using magnetic CD4 microbeads (Miltenyi Biotech, San Diego, CA) with a magnet according to the manufacturer's instructions. The purity of the CD4^+^ T-cells after sorting was >95%. The cDNA library construction, library purification, and transcriptome sequencing were implemented according to the Shanghai Genergy Biotechnology Sequencing Company's instructions.

### 2.8. Statistical Analysis

All statistical analyses were performed using GraphPad Prism 6 (GraphPad Software, San Diego, CA, USA). The statistical significance between values was determined by 2-tailed unpaired Student's *t*-test or one-way ANOVA with Dunnett's post hoc test when samples were not distributed normally. All data represent the mean ± SEM. ^∗^*P* < 0.05, ^∗∗^*P* < 0.01, ^∗∗∗^*P* < 0.001, and ^∗∗∗∗^*P* < 0.0001, ns: not significant.

## 3. Results

### 3.1. The Accumulation of Human MDSCs Is Remarkably Increased in Skin Lesions of Psoriasis Patients

**Figure 1:**
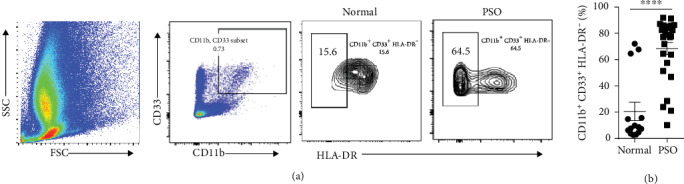
The accumulation of human MDSCs is remarkably increased in skin lesions of psoriasis patients. Representative flow cytometry panels for quantification of the accumulation of human MDSCs (CD11b^+^ CD33^+^ HLA-DR^−^) in skin lesions of patients with psoriasis (PSO, *n* = 27) and healthy control subjects (Normal, *n* = 17). Statistical analysis data is shown in (b). ^∗∗∗∗^*P* < 0.0001, 2-tailed unpaired Student's *t*-test was used.

Recently, the accumulation of MDSCs has been observed in the peripheral blood or spleen of murine models in autoimmune disorders such as SLE and RA, which are positively related to disease severity [12, 13, 15] and the number of MDSCs has been found expanded in psoriasis patients [16, 19, 21]. To study the relationship between psoriasis and MDSCs, we analyzed the population of MDSCs in skin lesions of psoriasis patients by flow cytometry. The human MDSCs have been identified with CD11b^+^ CD33^+^ HLA-DR^−^ [7, 10]. The details of patients for subjects participating in this study are shown in Table 1. We found that the accumulation of human MDSCs (CD11b^+^ CD33^+^ HLA-DR^−^) is remarkably increased in psoriatic skin lesions compared with healthy controls (Figure 1), indicating there is a correlation between psoriasis and the accumulation of MDSCs, to some extent.

### 3.2. MDSC Inhibitor (Gemcitabine) Significantly Attenuates IMQ-Induced Psoriasis-Like Skin Inflammation through Downregulating Th17 and Treg Cells

**Figure 2:**
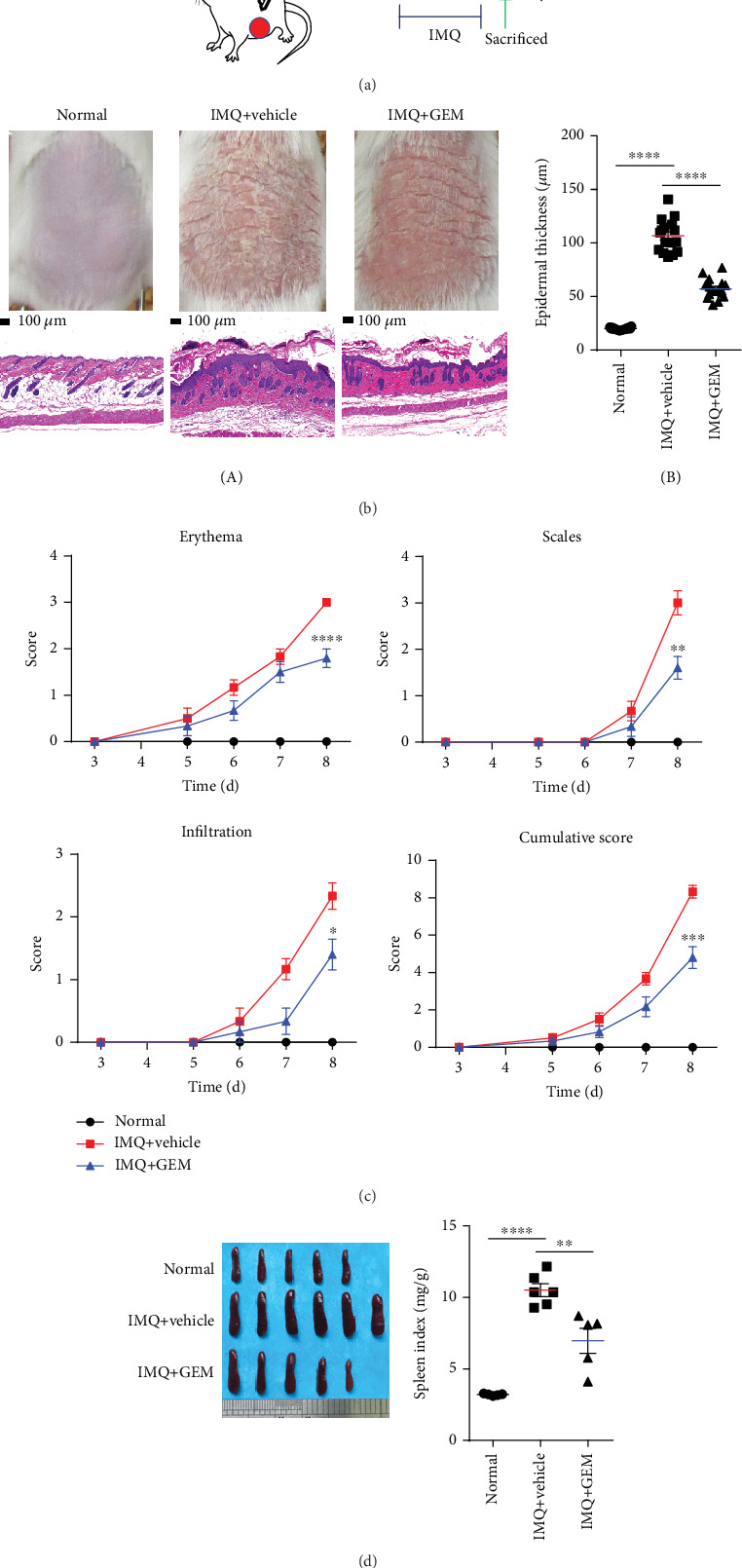
Gemcitabine significantly attenuates IMQ-induced psoriasis-like skin inflammation. (a) The specific drug use scheme. (b) The skin lesions and H&E staining of the back skin derived from mice injected intraperitoneally with vehicle (IMQ+vehicle) or Gemcitabine (IMQ+GEM) or untreated (Normal) (one representative mouse from each group is presented, *n* = 5–6 mice per group). Scale bars: 100 *μ*m. Statistical analysis data is shown in (B). (c) The PASI score of mice in 3 groups. (d) The spleens and statistical analysis of the spleen index (mg/g) of mice among 3 groups. All results are representative of at least 3 independent experiments. ^∗^*P* < 0.05, ^∗∗^*P* < 0.01, ^∗∗∗^*P* < 0.001, and ^∗∗∗∗^*P* < 0.0001; ns: not significant. One-way ANOVA with Dunnett's post hoc test was used.

**Figure 3:**
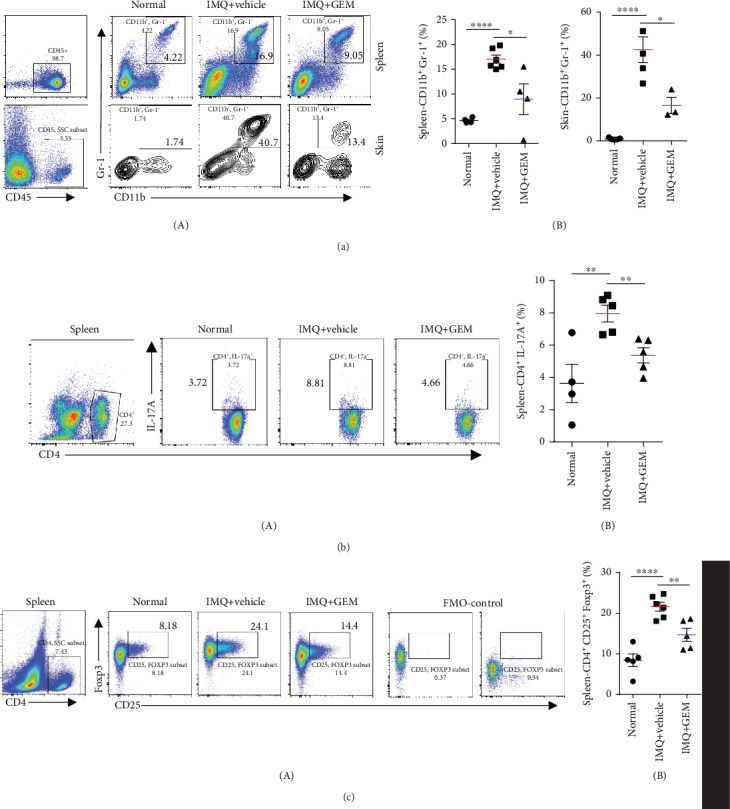
MDSC inhibitor (Gemcitabine) attenuates IMQ-induced psoriasis-like skin inflammation through downregulating Th17 and Treg cells. (a) Representative flow cytometry panels for quantification of MDSCs in spleen and skin lesions of BALB/c mice (*n* = 3–6 mice per group). CD11b^+^ Gr-1^+^ cells were selected from CD45^+^ cells. Statistical analysis data is shown in (B). (b) Representative flow cytometry panels for quantification of splenic Th17 cells of BALB/c mice (*n* = 4–6 mice per group). Statistical analysis data is shown in (B). (c) Representative flow cytometry panels for quantification and FMO-control of splenic Treg cells of BALB/c mice (*n* = 5–6 mice per group). Statistical analysis data is shown in (B). ^∗^*P* < 0.05, ^∗∗^*P* < 0.01, ^∗∗∗^*P* < 0.001, and ^∗∗∗∗^*P* < 0.0001; ns: not significant. One-way ANOVA with Dunnett's post hoc test was used.

Although the number of MDSCs has been found elevated in both skin lesions and peripheral blood, the effect of MDSCs on the pathogenesis of psoriasis remains to be elucidated. Gemcitabine (GEM) is well known to be an inhibitor of MDSCs, which reduces the accumulation of MDSCs with no significant influence on other immune cells such as T, B cells, NK cells, and macrophages [22]. Therefore, we treated mice with GEM to study the relationship between MDSCs and psoriasis. The specific drug use scheme is shown in Figure 2(a). The murine MDSCs have been characterized by CD11b^+^ and Gr-1^+^ [7, 10], and we found that IMQ treatment significantly induces psoriasis-like skin inflammation as well as the accumulation of MDSCs in spleen and skin lesions (Figures 2(b) and 3(a)). As expected, GEM treatment significantly reduces IMQ-induced accumulation of MDSCs in skin lesions and spleen (Figure 3(a)), therefore alleviating the phenotype of IMQ-induced psoriasis-like skin inflammation (Figure 2(b)) based on the Psoriasis Area and Severity Index (PASI) score (Figure 2(c)). In addition, GEM treatment markedly decreases IMQ-induced epidermal thickening and inhibited splenomegaly compared with the vehicle on day 6 after IMQ application for 5 consecutive days topically (Figures 2(b) and 2(d)). Moreover, GEM treatment remarkably decreases IMQ-mediated infiltration of Th17 and Treg cells in the spleen (Figures 3(b) and 3(c)), indicating depletion of MDSCs by GEM abrogates IMQ-induced psoriasis-like skin inflammation such as erythema, skin thickening, scaling, and the infiltration of Th17 and Treg cells.

### 3.3. The Effect of Depletion of MDSCs on Gene Expression Profiles of CD4^+^ T-Cells

**Figure 4:**
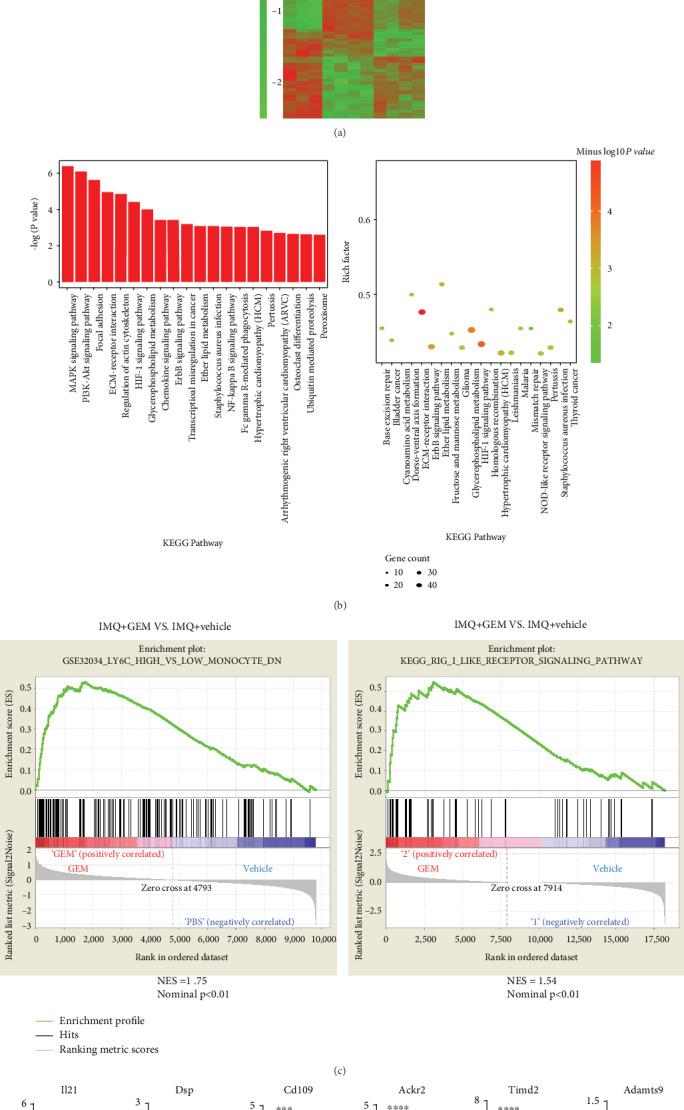
The effect of depletion of MDSCs on gene expression profiles of CD4^+^ T-cells. (a) Heatmap illustrates the expression levels of differentially expressed genes among the three groups. (b) The KEGG pathway showed the top significant function enriched pathway among differentially expressed genes. (c) GSEA enrichment plots for the immunologic signatures and KEGG pathways between IMQ+GEM versus IMQ+vehicle. Results were calculated from three subjects analyzed in the same batch. Normalized enrichment score (NES) and nominal *P* value are shown below each plot. (d) Identification of the differentially screened genes by qRT-PCR (*n* = 4–6 mice per group). The results were normalized to Gapdh. ^∗^*P* < 0.05, ^∗∗^*P* < 0.01, ^∗∗∗^*P* < 0.001, and ^∗∗∗∗^*P* < 0.0001; ns, not significant. One-way ANOVA with Dunnett's post hoc test was used.

To further investigate the detailed effect of MDSCs on CD4^+^ T-cells, we performed the RNA-seq technique to analyze transcriptional alteration of CD4^+^ T-cells after depletion of MDSCs by GEM. We found that 40 genes were upregulated, and 198 genes were downregulated after GEM treatment (Figure 4(a)). KEGG pathway analysis exhibited that the top significant differential expression of enriched pathways include the MAPK signaling pathway, PI3K-Akt signaling pathway, ECM-receptor interaction, and HIF-1 signaling pathway (Figure 4(b)). Next, we also performed gene-set-enrichment analysis (GSEA), which showed those differentially expressed genes are enriched in LY6C_HIGH_VS_LOW_MONOCYTE_DN and RIG_I_LIKE_RECEPTOR_SIGNALING_PATHWAY (Figure 4(c)). Thus, the results of GSEA based on transcriptional profiling of those splenic CD4^+^ T-cells revealed enriched genes downregulated in Ly6C monocytes and the RIG-I-like receptor signaling pathway was more activated in CD4^+^ T-cells after depleting MDSCs by GEM. Furthermore, we validated the expression of IL-21, Dsp, Cd109, Ackr2, Timd2, and Adamts9 in GEM-treated mice through qRT-PCR (Figure 4(d)), which have been documented to regulate Th17-cell differentiation (IL-21) [23, 24], Th1/Th17 immune skewing (Dsp) [25], T-cell activation (CD109, Timd2) [26–28], inflammatory T-cell chemotaxis (Ackr2) [29], and immune suppression (Adamts9) [30].

### 3.4. Neutralizing IL-21R In Vivo Inhibits IMQ-Induced Epidermal Thickening, Cutaneous MDSC Infiltration, and Splenic Th17 Infiltration

**Figure 5:**
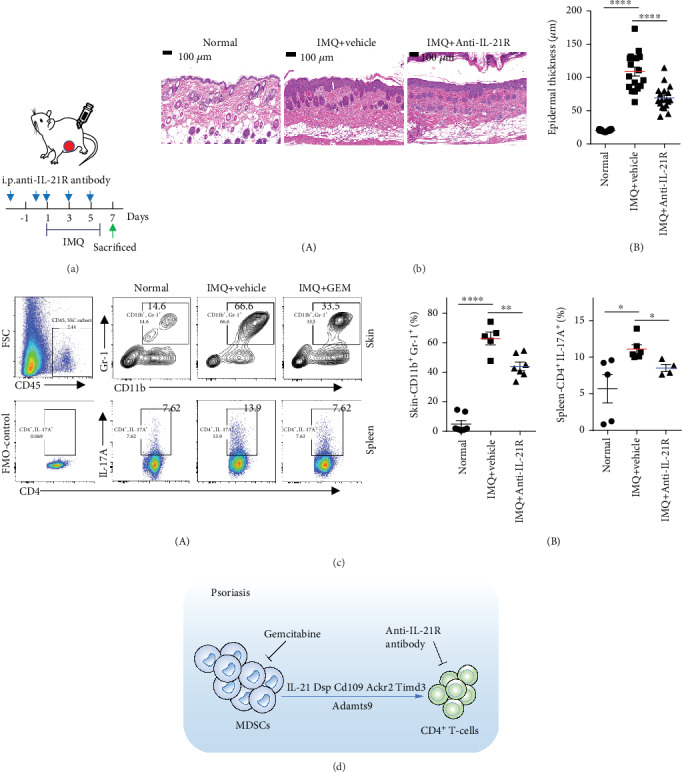
Neutralizing IL-21R in vivo inhibits IMQ-induced epidermal thickening, cutaneous MDSCs infiltration, and splenic Th17 infiltration. (a) Schematic illustration of the experimental setup. (b) The H&E staining of the back skin derived from mice injected intraperitoneally with vehicle (IMQ+vehicle) or anti-mouse IL-21R antibody (IMQ+anti-IL-21R) or untreated (Normal) (one representative mouse is presented, *n* = 4–7 mice per group). Scale bars: 100 *μ*m. Statistical analysis data is shown in (B). (c) Representative flow cytometry panels for quantification and FMO-control of cutaneous MDSCs and splenic Th17 cells of BALB/c mice (*n* = 4–7 mice per group). CD11b^+^ Gr-1^+^ cells were selected from CD45^+^ cells. Statistical analysis data is shown in (B). (d) Schematic illustration of targeting MDSCs attenuates IMQ-induced psoriasis-like skin inflammation. ^∗^*P* < 0.05, ^∗∗^*P* < 0.01, ^∗∗∗^*P* < 0.001, and ^∗∗∗∗^*P* < 0.0001; ns, not significant. One-way ANOVA with Dunnett's post hoc test was used.

Evidence revealed that IL-21 is highly expressed in the psoriatic skin lesions, which stimulates the proliferation of keratinocytes [31]. Moreover, IL-21 is well known to be related to immune diseases and regulates the differentiation of CD4^+^ T-cells [32]. IL-21R, a receptor for IL-21, is a class I cytokine heterodimeric receptor, which mainly expressed on lymphoid cells such as circulating T-cells, B cells, NK cells, and nonlymphocytic cells and tissues including keratinocytes [31]. To verify the role of IL-21 in the progression of psoriasis, we administrated the anti-IL-21R antibody to neutralize the IL-21 signaling pathway through IMQ-induced psoriasis-like BALB/c mouse models. The experimental design scheme is shown in Figure 5(a). The result of H&E staining and quantification showed that neutralizing IL-21R *in vivo* inhibits IMQ-induced epidermal thickening (Figure 5(b)). Moreover, neutralizing IL-21R with anti-mouse IL-21R antibody significantly reduces IMQ-mediated accumulation of MDSCs in skin lesions and splenic Th17 cells (Figure 5(c)), indicating targeting IL-21 is a therapeutic approach for psoriasis.

## 4. Discussion

Psoriasis has been documented to be a T-cell-mediated chronic inflammatory disease [2, 33]. The IL-23/IL-17A-Th17 axis has a crucial role in the development of psoriasis [2, 34, 35]. IL-23, secreted by DCs or KCs, facilitates Th17 differentiation which produces proinflammatory cytokines including IL-17A, IL-17F, IL-6, IL-21, and IL-22, resulting in the infiltration of Th17 and high levels of Th17-mediated proinflammatory cytokines in skin lesions and peripheral blood of psoriasis patients [2, 36, 37]. The Treg cells, constitutively expressing Foxp3 (the master transcriptional factor of Treg cells), are believed to maintain immune homeostasis through suppressing the function of other lymphocytes such as Th1, Th2, and Th17, resulting in inhibition of immune and inflammatory responses [38–40].

Although the role of Treg cells in psoriasis has not been fully elucidated, studies showed that numbers of Treg cells are upregulated in psoriatic skin lesions [41–44] or peripheral blood [39, 43, 44] of psoriasis patients or murine models [45]. In addition, evidence has indicated that Foxp3^+^ Treg cells can converse into inflammation-associated Th17 cells under proinflammatory conditions both in psoriasis [18, 46, 47] and in rheumatoid arthritis (RA) [48]. And there is a positive correlation between Treg cells and Th17 cells in psoriasis [43]. Moreover, accumulating studies demonstrated that the polarization of Th17 cells has been related to the induction of Foxp3^+^ Treg cells [18, 46, 49].

MDSCs are known to be a heterogeneous population of progenitor and immature myeloid cells derived from different stages and have essential roles for regulating the function of Th17 and Treg cells. The expanded MDSCs enhance the differentiation of naive CD4^+^ T-cell precursors into Th17 cells and are positively correlated with disease severity of SLE and RA patients as well as their murine models [12–15]. Our results showed that GEM, an MDSC inhibitor, inhibits IMQ-induced epidermal thickening and the accumulation of Th17, Treg cells, and MDSCs (Figure 3). Furthermore, we investigated the effect of depleting MDSCs by GEM treatment on gene expression profiles of CD4^+^ T-cells and the results exhibited that IMQ-induced IL-21 expression has been dramatically suppressed by GEM treatment (Figures 4(a) and 4(d)). IL-21 is highly expressed in skin lesions and peripheral blood of psoriasis patients, which is required for epidermal hyperplasia and Th17-cell polarization [23, 24, 31, 50]. And it was reported that IL-21 promotes psoriatic inflammation by inducing an imbalance of Th17 and Treg cells [47]. Consistent with those results, neutralizing IL-21R by its antibody abrogates IMQ-induced epidermal thickening, MDSC migration, and Th17 infiltration (Figure 5), indicating IL-21 may be a potential therapeutic target for psoriasis treatment.

Still, there are limitations in the present study which merit consideration. For example, our intervention to deplete MDSCs by GEM was at the animal level; thus, our hypothesis needs further investigations to verify. In addition, we have noticed the numerous side effects of GEM during application in humans, such as the dose-limiting toxicity (myelosuppression, thrombocytopenia, and anemia) and the minimal nonhematologic toxicity (nausea, shortness of breath, mouth sores, diarrhea, neuropathy, hair loss, etc.) [51], which may limit the chance of GEM being a useful therapy in psoriasis patients. However, we verified the significant anti-inflammatory effects by depleting MDSCs. Despite the severe side effects of Gemcitabine, we still can conclude that targeting MDSCs is a potential strategy for antipsoriasis therapy.

In summary, our study provided evidence that MDSCs play a proinflammatory role in IMQ-induced psoriasis-like skin inflammation and regulating the infiltration of CD4^+^ T-cells (Figure 5(d)). Depleting MDSC by its inhibitor (Gemcitabine) significantly suppresses the IMQ-mediated psoriatic phenotype as well as the accumulation of Th17 and Treg cells. Furthermore, we identified and validated the transcriptional expression changes of genes including IL-21 and Timd2 on CD4^+^ T-cells of GEM-treated mouse models, which are involved in Th17-cell differentiation or T-cell activation. Neutralizing IL-21R by antibody reduces IMQ-induced epidermal thickening through downregulating the infiltration of MDSCs and Th17 cells (Figure 5(d)), suggesting the accumulation of MDSCs exerts important function for the pathogenesis of psoriasis and IL-21 may be a potential therapeutic target in psoriasis.

## 5. Conclusions

Targeting myeloid-derived suppressor cells is a novel strategy for antipsoriasis therapy. IL-21 may be a potential therapeutic target in psoriasis.

## Figures and Tables

**Table 1:** Demographics of psoriasis patients and healthy control subjects.

Characteristics	Psoriasis patients	Healthy controls
Number of analyzed patients	27	17
Age in years, mean ± SD	35 ± 11	
Gender	66% males, 34% females	
Race/ethnicity	100% Chinese	
PASI score, mean(range) ± SD	4.4(0–10.5) ± 2	N/A

Abbreviations: N/A: not applicable; PASI: Psoriasis Area and Severity Index. ^1^Some patients were treated with multiple therapies. ^2^One patient (out of 27) had concurrent palmoplantar psoriasis. And one patient (out of 27) had concurrent arthropathic psoriasis.

## Data Availability

The RNA-seq data that support the findings of this study have been deposited in the CNSA (https://db.cngb.org/cnsa/) of CNGBdb with accession number CNP0001133.

## Abbreviations

KCs: Keratinocytes
MDSCs: Myeloid-derived suppressor cells
IMQ: Imiquimod
GEM: Gemcitabine
Th17: IL-17A-producing cells
Treg cells: Regulatory T-cells.

## Supplementary Materials

Supplementary Materials Table S1: a list of the primer for qPCR. Table S2: a list of the antibodies for flow cytometry.

## References

[1] Boehncke W. H., Schon M. P. (2015). Psoriasis. *Lancet*.

[2] Perera G. K., Di Meglio P., Nestle F. O. (2012). Psoriasis. *Annual Review of Pathology*.

[3] Fan Q., Gu D., Liu H. (2014). Defective TGF-*β* signaling in bone marrow-derived cells prevents hedgehog-induced skin tumors. *Cancer Research*.

[4] Kapanadze T., Gamrekelashvili J., Ma C. (2013). Regulation of accumulation and function of myeloid derived suppressor cells in different murine models of hepatocellular carcinoma. *Journal of Hepatology*.

[5] Lowes M. A., Suarez-Farinas M., Krueger J. G. (2014). Immunology of psoriasis. *Annual Review of Immunology*.

[6] Stumpfova M., Ratner D., Desciak E. B., Eliezri Y. D., Owens D. M. (2010). The immunosuppressive surface ligand CD200 augments the metastatic capacity of squamous cell carcinoma. *Cancer Research*.

[7] Gabrilovich D. I., Nagaraj S. (2009). Myeloid-derived suppressor cells as regulators of the immune system. *Nature Reviews Immunology*.

[8] Gabrilovich D. I., Ostrand-Rosenberg S., Bronte V. (2012). Coordinated regulation of myeloid cells by tumours. *Nature Reviews Immunology*.

[9] Veglia F., Perego M., Gabrilovich D. (2018). Myeloid-derived suppressor cells coming of age. *Nature Immunology*.

[10] Poschke I., Kiessling R. (2012). On the armament and appearances of human myeloid-derived suppressor cells. *Clinical Immunology*.

[11] Mengos A. E., Gastineau D. A., Gustafson M. P. (2019). The CD14+HLA-DRlo/neg monocyte: an immunosuppressive phenotype that restrains responses to cancer immunotherapy. *Frontiers in Immunology*.

[12] Guo C., Hu F., Yi H. (2015). Myeloid-derived suppressor cells have a proinflammatory role in the pathogenesis of autoimmune arthritis. *Annals of the Rheumatic Diseases*.

[13] Wu H., Zhen Y., Ma Z. (2016). Arginase-1-dependent promotion of TH17 differentiation and disease progression by MDSCs in systemic lupus erythematosus. *Science Translational Medicine*.

[14] Yi H., Guo C., Yu X., Zuo D., Wang X. Y. (2012). Mouse CD11b+Gr-1+ myeloid cells can promote Th17 cell differentiation and experimental autoimmune encephalomyelitis. *Journal of Immunology*.

[15] Zhang H., Wang S., Huang Y. (2015). Myeloid-derived suppressor cells are proinflammatory and regulate collagen-induced arthritis through manipulating Th17 cell differentiation. *Clinical Immunology*.

[16] Cao L. Y., Chung J. S., Teshima T. (2016). Myeloid-derived suppressor cells in psoriasis are an expanded population exhibiting diverse T-cell-suppressor mechanisms. *The Journal of Investigative Dermatology*.

[17] Oka T., Sugaya M., Takahashi N. (2017). CXCL17 attenuates imiquimod-induced psoriasis-like skin inflammation by recruiting myeloid-derived suppressor cells and regulatory T cells. *Journal of Immunology*.

[18] Soler D. C., McCormick T. S. (2011). The dark side of regulatory T cells in psoriasis. *The Journal of Investigative Dermatology*.

[19] Soler D. C., Young A. B., Fiessinger L. (2016). Increased, but functionally impaired, CD14(+) HLA-DR(-/low) myeloid-derived suppressor cells in psoriasis: a mechanism of dysregulated T cells. *The Journal of Investigative Dermatology*.

[20] Peng C., Zhang S., Lei L. (2017). Epidermal CD147 expression plays a key role in IL-22-induced psoriatic dermatitis. *Scientific Reports*.

[21] Ilkovitch D., Ferris L. K. (2016). Myeloid-derived suppressor cells are elevated in patients with psoriasis and produce various molecules. *Molecular Medicine Reports*.

[22] Suzuki E., Kapoor V., Jassar A. S., Kaiser L. R., Albelda S. M. (2005). Gemcitabine selectively eliminates splenic Gr-1+/CD11b+ myeloid suppressor cells in tumor-bearing animals and enhances antitumor immune activity. *Clinical Cancer Research*.

[23] Yang L., Anderson D. E., Baecher-Allan C. (2008). IL-21 and TGF-beta are required for differentiation of human T(H)17 cells. *Nature*.

[24] Zhou L., Ivanov I. I., Spolski R. (2007). IL-6 programs T_H_-17 cell differentiation by promoting sequential engagement of the IL-21 and IL-23 pathways. *Nature Immunology*.

[25] Paller A. S., Czarnowicki T., Renert-Yuval Y. (2018). The spectrum of manifestations in desmoplakin gene (*DSP*) spectrin repeat 6 domain mutations: Immunophenotyping and response to ustekinumab. *Journal of the American Academy of Dermatology*.

[26] Haregewoin A., Solomon K., Hom R. C. (1994). Cellular expression of a GPI-linked T cell activation protein. *Cellular Immunology*.

[27] Kuchroo V. K., Umetsu D. T., DeKruyff R. H., Freeman G. J. (2003). The TIM gene family: emerging roles in immunity and disease. *Nature Reviews Immunology*.

[28] Lin M., Sutherland D. R., Horsfall W. (2002). Cell surface antigen CD109 is a novel member of the alpha(2) macroglobulin/C3, C4, C5 family of thioester-containing proteins. *Blood*.

[29] Shams K., Wilson G. J., Singh M. (2017). Spread of psoriasiform inflammation to remote tissues is restricted by the atypical chemokine receptor ACKR2. *The Journal of Investigative Dermatology*.

[30] Du W., Wang S., Zhou Q. (2013). *ADAMTS9* is a functional tumor suppressor through inhibiting AKT/mTOR pathway and associated with poor survival in gastric cancer. *Oncogene*.

[31] Caruso R., Botti E., Sarra M. (2009). Involvement of interleukin-21 in the epidermal hyperplasia of psoriasis. *Nature Medicine*.

[32] Long D., Chen Y., Wu H., Zhao M., Lu Q. (2019). Clinical significance and immunobiology of IL-21 in autoimmunity. *Journal of Autoimmunity*.

[33] Mak R. K., Hundhausen C., Nestle F. O. (2009). Evolucion en la comprension de la inmunopatologia de la psoriasis. *Actas Dermo-Sifiliográficas*.

[34] Wu R., Zeng J., Yuan J. (2018). MicroRNA-210 overexpression promotes psoriasis-like inflammation by inducing Th1 and Th17 cell differentiation. *The Journal of Clinical Investigation*.

[35] Zhu H., Lou F., Yin Q. (2017). RIG-I antiviral signaling drives interleukin-23 production and psoriasis-like skin disease. *EMBO Molecular Medicine*.

[36] Haider A. S., Lowes M. A., Suarez-Farinas M. (2008). Identification of cellular pathways of "type 1," Th17 T cells, and TNF- and inducible nitric oxide synthase-producing dendritic cells in autoimmune inflammation through pharmacogenomic study of cyclosporine A in psoriasis. *Journal of Immunology*.

[37] Lee E., Trepicchio W. L., Oestreicher J. L. (2004). Increased expression of interleukin 23 p19 and p40 in lesional skin of patients with psoriasis vulgaris. *The Journal of Experimental Medicine*.

[38] Lehmann J., Huehn J., de la Rosa M. (2002). Expression of the integrin alpha Ebeta 7 identifies unique subsets of CD25+ as well as CD25- regulatory T cells. *Proceedings of the National Academy of Sciences of the United States of America*.

[39] Sugiyama H., Gyulai R., Toichi E. (2004). Dysfunctional blood and target tissue CD4+CD25high regulatory T cells in psoriasis: mechanism underlying unrestrained pathogenic effector T cell proliferation. *Journal of Immunology*.

[40] Yun W. J., Lee D. W., Chang S. E. (2010). Role of CD4CD25FOXP3 regulatory T cells in psoriasis. *Annals of Dermatology*.

[41] Cordoro K. M., Hitraya-Low M., Taravati K. (2017). Skin-infiltrating, interleukin-22-producing T cells differentiate pediatric psoriasis from adult psoriasis. *Journal of the American Academy of Dermatology*.

[42] Sanchez Rodriguez R., Pauli M. L., Neuhaus I. M. (2014). Memory regulatory T cells reside in human skin. *The Journal of Clinical Investigation*.

[43] Zhang L., Li Y., Yang X. (2016). Characterization of Th17 and FoxP3(+) Treg cells in paediatric psoriasis patients. *Scandinavian Journal of Immunology*.

[44] Zhang L., Yang X. Q., Cheng J., Hui R. S., Gao T. W. (2010). Increased Th17 cells are accompanied by FoxP3(+) Treg cell accumulation and correlated with psoriasis disease severity. *Clinical Immunology*.

[45] Hartwig T., Zwicky P., Schreiner B. (2018). Regulatory T Cells Restrain Pathogenic T Helper Cells during Skin Inflammation. *Cell Reports*.

[46] Bovenschen H. J., van de Kerkhof P. C., van Erp P. E., Woestenenk R., Joosten I., Koenen H. J. (2011). Foxp3+ regulatory T cells of psoriasis patients easily differentiate into IL-17A-producing cells and are found in lesional skin. *The Journal of Investigative Dermatology*.

[47] Shi Y., Chen Z., Zhao Z. (2019). IL-21 induces an imbalance of Th17/Treg cells in moderate-to-severe plaque psoriasis patients. *Frontiers in Immunology*.

[48] Komatsu N., Okamoto K., Sawa S. (2014). Pathogenic conversion of Foxp3+ T cells into TH17 cells in autoimmune arthritis. *Nature Medicine*.

[49] Yang X. O., Nurieva R., Martinez G. J. (2008). Molecular antagonism and plasticity of regulatory and inflammatory T cell programs. *Immunity*.

[50] Wang Y., Wang L. L., Yang H. Y., Wang F. F., Zhang X. X., Bai Y. P. (2016). Interleukin-21 is associated with the severity of psoriasis vulgaris through promoting CD4+ T cells to differentiate into Th17 cells. *American Journal of Translational Research*.

[51] Abbruzzese J. L., Grunewald R., Weeks E. A. (1991). A phase I clinical, plasma, and cellular pharmacology study of gemcitabine. *Journal of Clinical Oncology*.

