# Supplementary material for: Targeting Myeloid-Derived Suppressor Cells Is a Novel Strategy for Anti-Psoriasis Therapy
Source: Mediators Inflamm. 2020 Jun 28;2020:8567320. doi: 10.1155/2020/8567320 (PMC7338977; doi:10.1155/2020/8567320)
Supplement: Supplementary Materials — Table S1: a list of the primer for qPCR. Table S2: a list of the antibodies for flow cytometry. [file 8567320.f1.pdf]

Supplementary data

Supplementary table S1. A list of the primer for qPCR.

Gene	Species		Primer sequence
Il-21	mice	Forward	TGGATCCTGAACTTCTATCAGC
		Reverse	CACGAGGTCAATGATGAATGTC
Cd109	mice	Forward	TCTCGTTCTTCCGGCACTACCTC
		Reverse	TTCCTGCTTCGGCTTGTAGAATGC
Dsp	mice	Forward	AGGACCGTGAAGGATCAGGACATC
		Reverse	TCCTCCTCCAGCATCTTCCTCTTG
Timd2	mice	Forward	AATCTCTGAGCTTCGTTGCCTTCC
		Reverse	CTGGTCTTCACATCTGGTCCGTTC
Ackr2	mice	Forward	TCCATCTACGACTACGACTACT
		Reverse	AGGGCATAGTCACTACAAACAA
Adamts9	mice	Forward	GAGTACAGCGGATCGGACAATGTG
		Reverse	CACGGACAACACCTGAAGGAGAAG
Gapdh	mice	Forward	AACTTTGGCATTGTGGAAGG
		Reverse	ACACATTGGGGGTAGGAACA

Supplementary table S2. A list of the antibodies for flow cytometry.

For mice	Source	For human	Source
Zombie Aqua™ Fixable Viability Dye	BioLegend	APC/Cy7 anti-human CD45	BioLegend
Trustain fcX anti-mouse CD16/32	BioLegend	PerCP/Cy 5.5 anti-human CD11b	BioLegend
APC/Cy7 anti-mouse CD45	BioLegend	APC anti-human CD33	BioLegend
PE/Cy7 anti-mouse/human CD11b	BioLegend	BV510 anti-human HLA-DR	BioLegend
PerCP/Cy 5.5 anti-mouse Gr-1	BioLegend		
PE Rat anti-mouse IL-17A	BD Pharmingen		
APC anti-mouse IFN-r	BioLegend		
FITC anti-mouse CD4	BioLegend		
Anti-mouse CD25 APC	eBioscience		
Anti-mouse/rat Foxp3 PE	eBioscience		
